# Structural and Functional Analysis of Crucial Protein Complex in Basal Cell Skin Carcinoma via Network Construction

**DOI:** 10.22086/gmj.v0i0.1271

**Published:** 2018-12-31

**Authors:** Mona Zamanian Azodi, Mostafa Rezaei Tavirani, Majid Rezaei Tavirani, Mohammad Rostami-Nejad

**Affiliations:** ^1^Student Research Committee, Proteomics Research Center, Shahid Beheshti University of Medical Sciences, Tehran, Iran; ^2^Proteomics Research Center, Shahid Beheshti University of Medical Sciences, Tehran, Iran; ^3^Faculty of Medicine, Iran University of Medical Sciences, Tehran, Iran; ^4^Gastroenterology and Liver Diseases Research Center, Research Institute for Gastroenterology and Liver Diseases, Shahid Beheshti University of Medical Sciences, Tehran, Iran

**Keywords:** Protein Interaction Maps, Gene Ontology, Protein Complex, Skin Neoplasm

## Abstract

**Background:**

The basal-cell carcinoma (BCC) as one of the most common type of skin cancers reported to have an increasing trend during past years. Molecular approaches can be useful to advance diagnosis and treatment goals in this cancer.

**Materials and Methods:**

In this sense, one of the recent popular investigations, protein-protein interaction network analysis (PPI), was applied in this study to better facilitate molecular view of BCC. Cytoscape v3.6.0 and its plugins analyzed and explored the topological and annotation features of the constructed network.

**Result:**

Among TP53, EGFR, AKT1, ERBB2, HRAS, and CCND1 as central agents of the network, five of them were also present in the first prominent cluster of the network in which considered for further analysis. It is suggested that there are significant related biological processes, actions, and expression changes for this highlighted cluster that may be related to BCC risk.

**Conclusion:**

Therefore, the studied complex of proteins may worth considering for clinical studies and therapeutic interventions after validating by related tests. What is more, among these genes, EBBR2 has more to offer and consequently with additional values.

## Introduction

Basal-cell carcinoma is the most widespread type of skin cancer in the U.S [1] with the high incidence in white populations [2]. The rate had been reported to be three and four times elevated during the past 30 years [3]. Risk factors corresponding to BCC, are skin color, genetics, immunosuppressive treatment, arsenic ingestion, and most importantly exposure to ultraviolet radiation [2]. Different treatment approaches are present for this type of cancer including surgery, radiotherapy, photodynamic therapy, topical fluorouracil, and imiquimod; however, surgery is the most effective one [4]. To find better treatment strategies, molecular studies can help. In this regard, molecular agents have an indispensable role in BCC reported by many studies [5]. There are various type of molecular investigations about skin cancer including genetics, genomics, proteomics, and metabolomics. One way to provide deeper understanding in this respect is to study proteins in a whole system through protein-protein interaction mapping. Protein interactions are the main part of processes taking place in a cell [6]. This bioinformatics approach applies interaction data from different sources for the protein set of interest. The interaction information is provided from integration of previous studies available from different datasets [6]. In a disease condition, these proteins that play central role in an interactome profile are more important than those without centrality features. The reason is, central proteins possessing values including degree and betweenness centralities can have vast an impact on the interacting system when becoming aberrant [7]. Therefore, by studying these key proteins, it is feasible to improve cancer diagnosis and treatments [8]. In addition, identifying protein clusters of a network of interactions could be exploit for detecting mechanisms of the disease. The disease mechanisms are driven by many biological processes in which are organized by the mentioned clusters of proteins known as protein complexes [9]. The protein complexes are the condense components of an interaction network that are predictable through different clustering algorithms such as MCODE [10]. Basal-cell carcinoma is not an exception for computational analysis. Data available from genomics and proteomics can be used for this approach to introduce better candidates for BCC namely biomarkers. Here, we used protein-protein interaction network and protein complex analysis to provide a preliminary introduction of the possible targets of BCC.

## Materials and Methods

Network analysis of carcinomas can be helpful to better understand the essential nodes of an interacting system. Here, by the application of protein-protein interaction network analysis, Cytoscape v3.6.0, it is aimed to detect crucial parts of the map [11]. At first the skin carcinoma was queried against STRING db, V10.5 (http://string-db.org/), Plug-in and 100 mostly related proteins in this malignancy was considered for the network construction via assigning interaction confidence score > 0.4. Network analysis was conducted in terms of centrality evaluation including degree (K) and betweenness centrality (BC). In this study, we considered 10% of total nodes with highest degree and betweenness centrality as hub and bottlenecks, respectively. After analyzing central nodes of the network, we continued analyzing the network for cluster constitution prediction. For this aim, the molecular complex detection (MCODE) plug-in was used to define the most condense parts of the network in terms of connections. MCODE is a clustering algorithm that detect dense parts of the PPI network. This application works in three stages including vertex weighting, complex identification, and complex processing via connectivity criteria [10]. The analysis continued by enrichment analysis of the significant cluster of interest via ClueGO app v.2.5.1 http://www.ici.upmc.fr/cluego/ [12]. This software creates network of terms or pathways as functional groups. The applied statistics for this analysis is kappa statistics. In this way, terms with similarity in their genes contributions are grouped together. Functional groups are defined based on kappa score ≥ 0.5 and term fusion was applied to reduce the abundancy for the biological process examination. The higher the kappa score, the higher the possibility that these terms group together. Grouping level: Min =2, Max =8 P-value correction method: Bonferroni stepdown. Enrichment/depletion test: two-sided (enrichment/depletion) test based on hypergeometric distribution. For identifying action type between genes of cluster 1, CluePedia was applied for this analysis. The source for action identification was from STRING Action File in CluePedia Panel. Three types of actions including expression, activation, and inhibition were assigned for this evaluation. The scoring was based on kappa statistics ranging from 0 to 1. The scoring system can be shown by specifying thickness for the corresponding action type. Moreover, expression profiling of the identified genes were also investigated through GEO Database and GEO2R. A study entitled, Expression data from mouse basal cell carcinoma (BCC) and normal epidermis, and BCC cell line treated with cyclopamine”, GEO accession number: GSE20065 (https://www.ncbi.nlm.nih.gov/geo/query/acc.cgi?acc=) GSE20065 were used for this procedure, in a way that the genes were queried against the differentially expressed genes among normal and BCC samples.

## Result

**Figure1:**
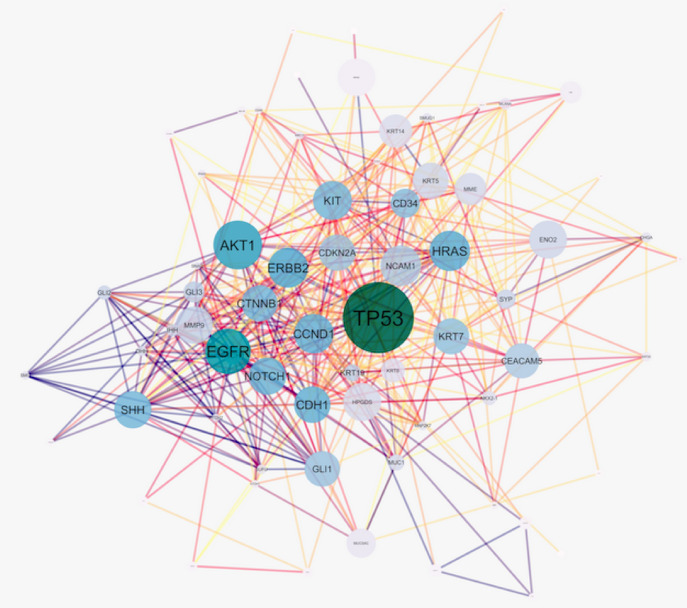
The main connected component of skin carcinoma related network is shown. The network is layout based on degree (K) value and betweenness centrality (BC). The color changes from dark to light express (K) value changes from high to low. Similarly, the node size changes from big to small shows (BC) value.

**Figure 2:**
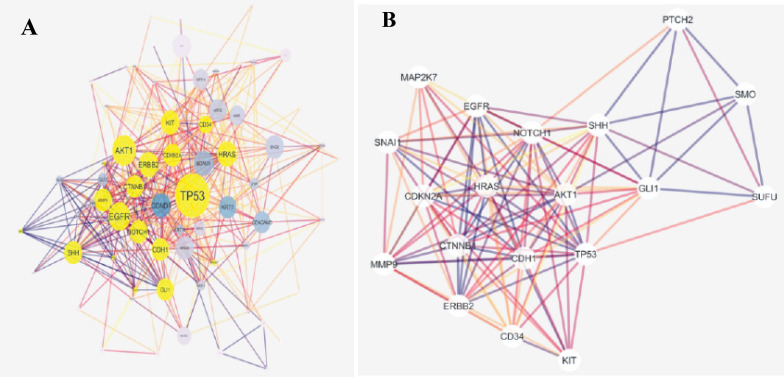
Cluster analysis of skin carcinoma network. The highest ranked cluster of our network is depicted. This complex consist of 19 nodes and 114 edges with the score of 12.7 **A:** cluster position in the network of skin carcinoma is highlighted in yellow color. **B:** the sub-network is shown individually. The statistical parameters are as follow: Degree Cutoff: 2, K-core=2, Node Score cut off= 0.2

**Figure 3:**
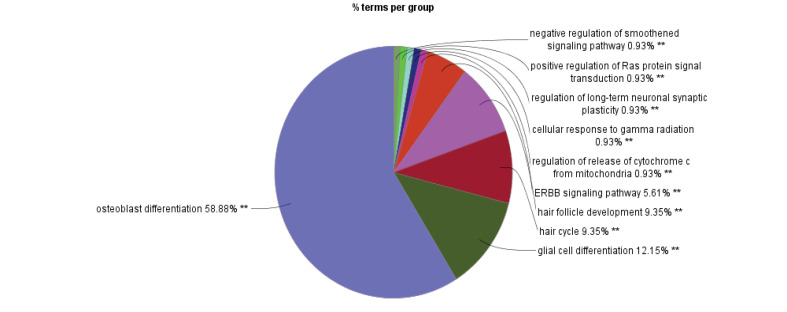
Chart overview of biological process analysis of the highlighted cluster of the BCC network via ClueGO plug-in. The functional groups are ordered based on percentage terms per group. The groups are named after the leading term and are assigned with different colors. Osteoblast differentiation (58.88%), glia cell differentiation (12.15%), hair cycle, and hair follicle development contain about 90% of the identified processes. Number of genes per term = 3, Percentage for the queried terms = 4 were considered.

**Figure 4:**
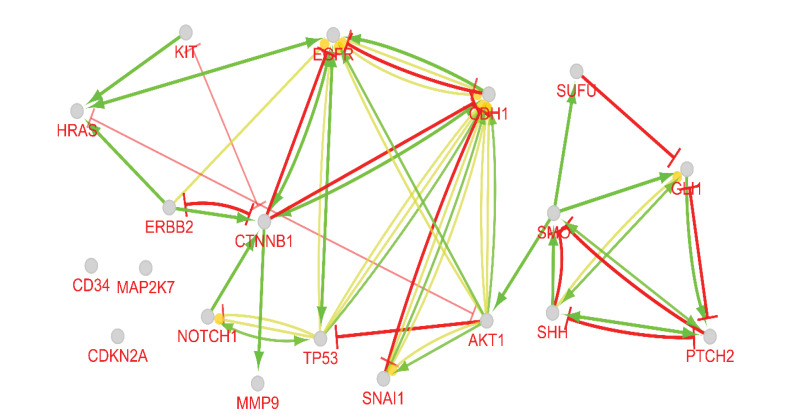
A CluePedia action view for the 19 genes including activation (green), expression (yellow), and inhibition (red). The edge thickness indicates the max and min scores, in which the cut off for these action scores was set to 0.5 (medium). While CD34, MAP2K7, and CDKN2A are not in the interaction, CTNNB1, EGFR, CDH1, and AKT1 show the most interacting features in this action network.

**Figure 5:**
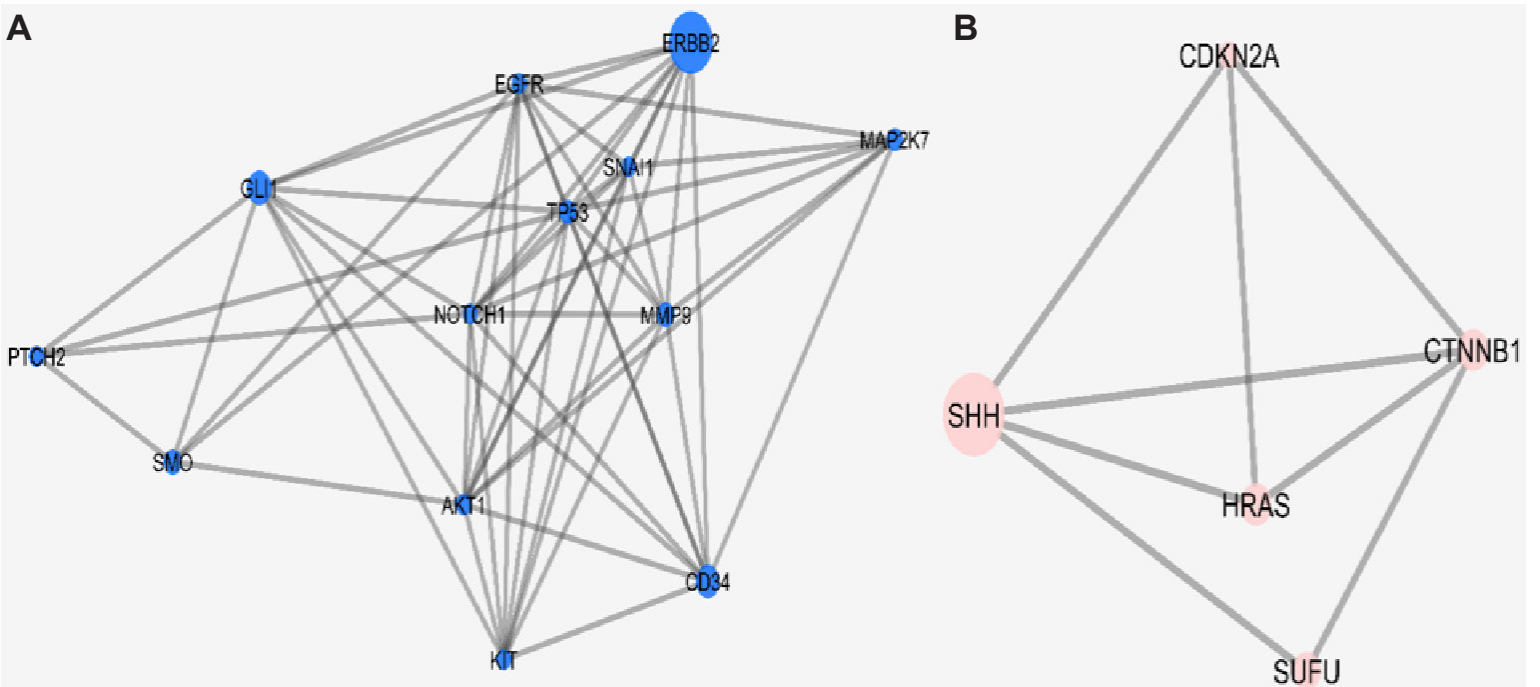
Gene expression analysis of 18 genes of high ranked cluster. The difference in expression values in the disease states are shown as node size changes. The bigger nodes are either more up-regulated or down-regulated in left** (A)** and right **(B)**, respectively. CDH1 is not included in this expression network because of not being present among expression genes. Most of the genes are up-regulated.

The original skin network consists of 100 nodes and 459 edges including 25 isolated nodes, 3-paired nodes, and a main connected component with 69 nodes and 456 edges. The edges number is not changed dramatically in which indicates that the most of the 31 nodes are isolated (see figure-1). The centrality analysis of genes is beneficial for detecting nodes with highest degree and betweenness centrality values that may be important in carcinoma onset and development. Here, results of topological analysis of the sub-network are presented in table-1. Protein complex identification can be helpful to better understand the vital clusters of an interaction network. These clusters of proteins are fundamental spots of the network functions [13]. All the hub-bottlenecks except CCND1 are present in the highest score complex of the network. This finding highlights the importance of the first cluster of the network. (See figure-2)The gene ontology based on biological processes of the top ranked cluster including 19 genes shows the most relevant groups of terms (See figure-3). Expression profile of the top ranked cluster is presented in the figure-4.Expression profile of the 18 genes among the 19 members of high ranked cluster is obtained from a gene expression study entitled “Expression data from mouse basal cell carcinoma (BCC) and normal epidermis, and BCC cell line treated with cyclopamine” from GEO. The logFC of the 18 genes was converted to FC and after normalization the related network was constructed (See suppl tables-1-3 and figure-5). The tables from 1 to 3 indicate expression profiling via GEO2R, normalization of up-regulated genes, and normalization of down-regulated genes.

**Table1:** Centrality statistical information of Skin Carcinoma network based on degree (K) and Betweeenness Centrality (BC) after analyzing with network analyzer. The nodes are ranked based on degree value. This calculation is obtained by considering 10% of nodes with highest K as hub-nodes and BC as bottlenecks. Finally, the common genes between these highest ranked values were chosen as hub-bottlenecks and are presented.

**Row**	**Gene Name**	** Degree**	**BC**
-1	TP53	49	0.24
2	EGFR	36	0.08
3	AKT1	33	0.1
4	ERBB2	30	0.04
5	HRAS	29	0.05
6	CCND1	28	0.04

## Discussion

Protein-protein interaction network analysis can be promising for introducing essential elements of an interactome scale of any kinds of diseases [8]. Recent advances in molecular analysis introduced some candidate agents that could be related to basal-cell carcinoma [14]. One of the useful methods for retrieving this information is through different sources of Cytoscape, network constructer and analyzer [11]. Detecting these nominates are accessible through STRING db via Cytoscape 3.6.1 v software. In this paper, 100 basal-cell carcinoma correlated genes are analyzed as a constructed network. The analysis shows that the skin PPI network is a scale free network consisting of some key proteins in terms of centrality. The topological identification of our network indicated that there are six proteins with this feature. The parameters used for this interpretation were degree and betweennness centrality considerations. The top ranked protein is TP53 with degree=49 and BC=0.24. This protein has been involved in many different types of cancers [15] and here it is also one of the key elements of the network. What is more, EGFR is also common in types of different cancers including skin cancer [16].

This protein is characterized as the second ranked hub-bottleneck with degree of 36 and BC of 0.08 in skin cancer network.

Other proteins (AKT, ERBB2, HRAS, and CCND1) are also reported to have a part in skin cancer [17-20]. About 83% of our central nodes are present in the highest ranked cluster of the skin carcinoma network. In other words, 26% of our cluster-1 nodes are from centrals of the main network. This fact indicates on the prominent role of the first cluster in the network and likewise the importance of the central nodes in the cluster-1 constitution. In addition to the central nodes the othe members of the identified cluster play crucial roles in BCC. For instance relation between PTCH gene and BCC is highlighted by Smyth *et al* [21]. To pinpoint more about the importance of the identified cluster and its vital role in skin cancer risk, the analysis continued by evaluation of different aspects of this complex including gene annotation, action type, and expression pattern identification. Gene ontology is used widely in interpretation of molecular aspects of different diseases [22,23]. The enrichment analysis showed that the top rated biological processes are Osteoblast differentiation and glia cell differentiation that may be interrupted in skin cancer. Action analysis in figure 4 indicated that there are some genes in a condense interactions while some are even not cooperating in any actions. The central nodes are the high interacted genes in the figure 4. They have strong control role in the gene expression regulation of the other genes of the analyzed cluster. Further analysis via expression identification may reveal more. As it is shown in figure 5, expression profiling of the queried genes of our cluster against an expression study, shows that there are some changes in expression levels of these genes. CDH1 was the only not identified gene among our investigated genes. All the genes showed significant expression differentiations (P≤ 0.05) in BCC except for SMO, NOTCH1, and SNAI1. ERBB2 with the fold change of 22.78 and SHH with the fold change of 17.26 are the most significant up-regulated and down-regulated genes. Both of these genes are in high interactions in terms of expression, activation, and inhibition. Additionally, ERBB2 is also among the hub-bottlenecks. Therefore, ERBB2 and SHH may play imperative role in BCC pathogenesis based on serial validation approaches in this study.

## Conclusion

In conclusion, the predicted protein complex with several vital properties can have a participation in skin cancer risk and should be considered for targeting evaluations. Moreover, ERBB2 may have an additional value considering its protein-protein interaction centrality, actions, and up-regulation in BCC. More investigation in this regard is suggested to support this finding.

## Conflict of Interest

The Authors declare no conflict of interest.
